# Computational Tools and Algorithms for Designing Customized Synthetic Genes

**DOI:** 10.3389/fbioe.2014.00041

**Published:** 2014-10-06

**Authors:** Nathan Gould, Oliver Hendy, Dimitris Papamichail

**Affiliations:** ^1^Department of Computer Science, The College of New Jersey, Ewing, NJ, USA; ^2^Department of Biology, The College of New Jersey, Ewing, NJ, USA

**Keywords:** computational biology, synthetic biology, gene design, codon bias, codon context

## Abstract

Advances in DNA synthesis have enabled the construction of artificial genes, gene circuits, and genomes of bacterial scale. Freedom in *de novo* design of synthetic constructs provides significant power in studying the impact of mutations in sequence features, and verifying hypotheses on the functional information that is encoded in nucleic and amino acids. To aid this goal, a large number of software tools of variable sophistication have been implemented, enabling the design of synthetic genes for sequence optimization based on rationally defined properties. The first generation of tools dealt predominantly with singular objectives such as codon usage optimization and unique restriction site incorporation. Recent years have seen the emergence of sequence design tools that aim to evolve sequences toward combinations of objectives. The design of optimal protein-coding sequences adhering to multiple objectives is computationally hard, and most tools rely on heuristics to sample the vast sequence design space. In this review, we study some of the algorithmic issues behind gene optimization and the approaches that different tools have adopted to redesign genes and optimize desired coding features. We utilize test cases to demonstrate the efficiency of each approach, as well as identify their strengths and limitations.

## Introduction

Expression of genes is fundamental to modern biotechnology. Expression is the process by which information from a gene is used in the synthesis of a functional gene product, most often a protein. Gene expression may be modulated at intermediate steps, including transcription, RNA splicing, translation, and post-translational modification of a protein. In this review, we will primarily concentrate on the process of translation, and the effect that synonymous mutations in a protein-coding gene confer to the expression of the corresponding protein. Working toward the objectives of synthetic biology, precise protein expression control has direct implications in improving heterologous expression, and in successfully designing and fine-tuning gene regulatory networks. Gene design has applications for metabolic engineering, particularly in biofuel production, where rate-limiting steps can be overcome through gene optimization (Wiedemann and Boles, 2008).

Advances in large-scale DNA synthesis, cloning, DNA sequencing, and design and assembly of building blocks to engineer biological systems have created unique opportunities for high-throughput experimentation toward broadening our understanding of gene structure, protein function, and genetic organization (Bugl et al., 2007; Czar et al., 2009a). Recent years have seen the development of a large number of computational tools that aim to enable life scientists to create their own synthetic genes and constructs. The first generation of design tools focused primarily on optimizing designs for manufacturability (i.e., oligos without local secondary structures and end repeats) instead of biological activity. But soon the oligo design process was separated from the gene optimization process, and new tools emerged that address the two processes separately.

This review focuses on software tools that aim to aid the redesign of existing genes for optimized protein expression, and the algorithms behind these tools, all viewed from a computational perspective. There exists a different set of tools that aid the design of synthetic DNA sequences based on functional blocks called genetic parts, which often utilize libraries of standard biological parts, such as Biobricks (Shetty et al., 2008). GenoCAD (Czar et al., 2009b) is one such tool that facilitates the construction of artificial DNA sequences by relying on formal design strategies and the notion of *grammars*, sets of rules describing the structure of DNA sequences. The genomic sequences that GenoCAD uses as biological parts are immutable, and no optimizations are applied in the creation of the constructs. Other circuit design tools include GEC (Pedersen and Phillips, 2009), TinkerCell (Chandran et al., 2009), and Clotho (Xia et al., 2011).

In Section “Gene Design Objectives and Algorithms,” we examine some of the most important objectives in synthetic gene design toward optimized expression, accompanied by a brief analysis of their computational complexity. Section “Gene Design Tools” is devoted to 11 currently available gene design tools incorporating the aforementioned objectives. We detail our experiences using these tools in optimizing the green fluorescent protein (GFP) gene *in silico* for heterologous expression in Section “Gene Design Tools in Practice,” followed by a brief discussion on the strengths and limitations of examined tools.

## Gene Design Objectives and Algorithms

Gene design software tools aim to guide the redesign of protein-coding genes using pre-defined features of interest, predominantly targeting improved protein expression, and simplified DNA sequence manipulation. In this section, we examine several criteria that have been traditionally used in optimizing gene expression, all of which are incorporated in one or more of the evaluated tools.

### Codon bias

In most species, synonymous codons are used at unequal frequencies. Codon usage bias is recognized as crucial in shaping gene expression and cellular function, affecting diverse processes from RNA processing to protein translation and protein folding. Rarely used codons have been associated with rare tRNAs and have been shown to inhibit protein translation, where favorable codons have the opposite effect, something that is particularly pronounced in prokaryotic organisms (Lithwick and Margalit, 2003). The process of substituting rare codons with favorable ones is referred to as codon optimization. Controlling codon bias, without considering other optimization objectives, to modulate translation rates is computationally easy, since it involves only certain synonymous substitutions to reach a desired distribution. The quantification of the effect though is much more difficult, due to the limited number of gene variants from only a handful of model organisms that have been evaluated in literature, limiting the reliability of gene expression predictions based on codon bias measures [such as the Codon Adaptation Index (CAI), described below]. Nevertheless, the use of particular codons through synonymous mutations has been shown to influence gene expression (Welch et al., 2009), and in certain cases to increase the expression of transgenes (genes expressed in a heterologous host) by more than 1000-fold (Gustafsson et al., 2004).

Numerous statistical methods have been proposed and used to analyze codon usage bias. Methods such as the *Frequency of optimal codons (Fop)* (Ikemura, 1981), the *CAI* (Sharp and Li, 1987), and the *tRNA adaptation index (tAI)* (Dos Reis et al., 2004) are used to quantify codon preferences toward over- or under-represented codons, and to predict gene expression levels, while methods such as the *Effective Number of codons (ENc)* and Shannon entropy from information theory (Suzuki et al., 2004) are used to measure codon usage evenness. *Relative Synonymous Codon Usage (RSCU)* (Sharp et al., 1986) and *Synonymous Codon Usage Order (SCUO)* (Wan et al., 2004) are additional examples in the latter category. Several of these methods have been used in studies examining the effect of codon bias on gene expression, often with little justification. CAI is the most prevalently used codon bias measure in pertinent literature, but that preference seems to be better explained by historical precedence rather than superior predictive power.

Optimization of codon bias as a singular objective is algorithmically straightforward and can be performed in linear time as a function of the sequence length. This is true for maximization or minimization toward any given codon bias measure (such as CAI, RSCU, ENc, etc.), as well as adoption/emulation of any given codon distribution, including the case when codon position assignments are performed randomly.

### Codon context bias

Gutman and Hatfield (1989) first noticed that codon pairs in prokaryotic genes exhibit another significant bias toward specific combinations. Further studies (Irwin et al., 1995) revealed that codon pair optimization influences translational elongation step times, but their functional significance was studied only in very small datasets. More recent work by Coleman et al. (2008), Mueller et al. (2010), and Coleman et al. (2011) who synthesized novel coding regions utilizing large-scale codon pair optimization and de-optimization, coupled with *de novo* synthesis of the constructs and *in vivo* experimentation, provided evidence of the influence codon pair bias has on translational efficiency. Several mathematical methods have been proposed for the study of codon context bias, including (Fedorov et al., 2002; Hooper and Berg, 2002; Shah et al., 2002; Boycheva et al., 2003; Moura et al., 2005; Coleman et al., 2008). Three of the gene design tools examined in this review provide functionality for controlling codon context, albeit no two tools share the same measure of codon context bias.

*Codon reuse* (or *autocorrelation*) is the grouping of synonymous codons in distinct regions of the mRNA transcript. Rare codons often form clusters, both in eukaryotic and prokaryotic genomes (Clarke et al., 2008), and codon reuse is considered to be a result of tRNA recycling, where a single tRNA is used, recharged, and used again for the same transcript (Godinic-Mikulcic et al., 2014). Genes that group their synonymous codons often express higher than those that do not (Cannarrozzi et al., 2010).

Optimization of codon context as a singular objective has linear time complexity as a function of sequence length. Optimization of codon pair bias with a fixed codon distribution is considerably harder, although polynomially time solvable. This latter problem can be reduced to a variation of the Traveling Salesperson Problem, which can be solved with a dynamic programing algorithm having a time bound of O(n^65^)^1^, where *n* is the length of the sequence being optimized (Mueller et al., 2010). As a consequence, all currently available tools that attempt to codon context optimize synthetic genes in conjunction with other objectives utilize metaheuristics such as simulated annealing or genetic algorithms. These heuristics do not guarantee an optimal solution, but limit the running time of the optimization procedure, while typically computing reasonable approximations.

### RNA secondary structure

The status of RNA in molecular biology has changed dramatically over the last decade. RNA molecules have been shown to function as key regulatory elements and participate in a wide spectrum of cellular processes (Dunham et al., 2004; Carninci et al., 2005; Kin et al., 2007). The number of known non-coding RNA sequences has surpassed 30 million, grouped into more than 1400 families (Gardner et al., 2009). Significant RNA structural elements in viruses, such as the CRE in poliovirus (Goodfellow et al., 2000, 2003), have distinct functions and their presence or absence is often critical in replication or translation. Such elements, in addition to well-described functional centers and catalytic cores of RNA molecules, will have many synthetic bioengineering uses and can be beneficial if inserted or removed from mRNA coding sequences, which can then assume catalytic activities and other secondary roles on top of their protein encoding functions.

Certain secondary structures within an mRNA can affect ribosome transit rates by temporarily stalling ribosomes while they attempt to unwind and translate through them (Buchan and Stansfield, 2007). Dense secondary structures have also been correlated with longer half-life of an RNA molecule in eukaryotic cells (Simmonds et al., 2004; Davis et al., 2008), a point of significance in the context of viral RNA. These results imply that by controlling the structure and free energy of an mRNA molecule, one may modulate its translation rate and persistence in a cell.

The most popular algorithms for RNA folding use empirically determined thermodynamic parameters (Freier et al., 1986; Serra and Turner, 1995) and dynamic programing for identifying the globally minimal energy structure; these are implemented in packages such as MFOLD (now UNAFold) (Zuker, 1989; Markham and Zuker, 2008) and ViennaRNA (Schuster et al., 1994), which are available as open source projects. The O(*n*^3^) time complexity of these algorithms render them of limited use when evaluating substantial numbers of RNA sequences, variants of genes, or when employing iterative methods to design customized synthetic genes. Cohen and Skiena (2003) have experimented with the most and least stable mRNA structures coding for a given protein (by applying only synonymous changes), having shown that the former can be computed in O(*n*^3^) time – the same complexity with RNA folding – and the latter is NP-hard^2^ to compute, implicating no algorithm is expected to exist that can generate an optimal solution for any realistically sized mRNA in reasonable time.

### Ribosomal binding site

The rate of protein synthesis in a cell depends on both the rates of translation initiation and elongation (Plotkin and Kudla, 2010). Translation initiation is often a critical rate-limiting step in protein production from mRNA, and is largely dependent on weak secondary mRNA structure in the 5′ untranslated region and near the start codon. A number of randomly mutated GFP transcripts were analyzed by Kudla et al. (2009), and they explained variation in expression with mRNA secondary structure in the first 47 nucleotides of the transcript. Espah Borujeni et al. (2014) provide direct evidence for this hypothesis by showing that secondary structure inhibits mRNA loading onto the 30s subunit and that partial unwinding of this mRNA usually occurs before accommodation by the ribosome.

As of today, there are three available tools that model translation initiation and aid the design of ribosomal binding site (RBS) sequences with desired translation initiation rates. These are *RBS Calculator* (Salis et al., 2009; Salis, 2011), *UTR Designer* (Sang et al., 2009; Seo et al., 2013), and *RBS Designer* (Na and Lee, 2010; Na et al., 2010). A recent review article analyzes these tools in detail (Reeve et al., 2014), and we will not delve into these tools’ functionality, since the design of an RBS sequence can be accomplished independently from the other characteristics of the protein-coding sequence.

### Restriction sites, hidden stop codons, and other motif avoidance

Restriction enzymes are laboratory agents that cleave DNA at specific motifs. Each occurrence of these motifs within a DNA sequence is called a *restriction site recognition site* or *restriction site*. Restriction sites that appear uniquely in a sequence allow for unambiguous cleavage, enabling DNA manipulation techniques such as subcloning, where a new sequence can be inserted between two different unique restriction sites. Thousands of restriction enzymes have been identified and indexed in databases such as REBASE (Roberts et al., 2009), and hundreds are available commercially. Most of the synthetic gene design tools we examine offer restriction site manipulation features, such as elimination and unique placement.

In prokaryotes, translation is initiated by hybridization of the 16S rRNA to the Shine–Dalgarno (SD) consensus sequence upstream of the start codon of the mRNA. It is thought that rare codon bias in N-terminal regions of prokaryotic genes may actually be a result of avoidance of SD-like sequences in the remainder of the gene. If SD consensus sequences are found in the rest of the gene, hybridization can actually occur again and cause translational pausing (Li et al., 2012). Thus, it is postulated that avoidance of SD-like codons is a driving force for codon bias, although it is expected that this SD-driven codon bias would only have implications for genes expressed in prokaryotic cells.

Several other factors have been posited to affect gene expression, including repeated nucleotides, potential polyadenylation sites (Pfarr et al., 1986), cryptic splice sites (Bukovac et al., 2008), nuclease cleavage sites (Smolke and Keasling, 2002), hidden stop codons (Seligmann and Pollock, 2004), and GC content (Kudla et al., 2006). Several of the gene design tools being reviewed address one or more of these factors, by providing simple mechanisms to remove the undesirable patterns. In particular, polyadenylation sites, nuclease cleavage sites, and hidden stop codons can be incorporated and/or eliminated with standard pattern inclusion/exclusion mechanisms that many tools offer. GC content modulation is usually performed by gene design tools offering multi-objective optimization.

Restriction site and other pattern placement/elimination is an NP-hard problem in its general form (Montes et al., 2010). Because most patterns have sufficient length to occur sparsely by chance, in conjunction with the small number of patterns that are usually targeted when designing genes, the problem of incorporating and eliminating patterns becomes trivial, and as a singular objective it has a linear time complexity as a function of sequence length. When pursued in conjunction with other objectives such as codon usage utilization, pattern elimination can lead to unattainable solutions, such as one amino acid – one codon designs with irremovable restriction sites. Most tools resolve such conundrums by prioritizing objectives, to the detriment of a universally optimum solution.

## Gene Design Tools

We have decided to investigate 11 software packages that are available at the time of this writing: DNAWorks, Jcat, Synthetic Gene Developer, GeneDesign, Gene Designer 2.0, OPTIMIZER, Visual Gene Developer, Eugene, mRNA Optimizer, Codon Optimization OnLine (COOL), and D-Tailor. All of these tools are available on the web and can be freely downloaded and/or used without any special permissions, requests, or elaborate procedures. The sole prerequisite for our selections is that the tools can be used to optimize DNA sequences that code for proteins, without altering the chain of amino acids. In Table 1, we summarize the gene design tools we examined, the web address where each tool can be found, and the corresponding publication where the software was first introduced.

**Table 1:** **Gene design tools**.

Gene design tool	Web URL	Reference
DNAWorks	http://helixweb.nih.gov/dnaworks/	Hoover and Lubkowski (2002)
Jcat	http://www.jcat.de/	Grote et al. (2005)
Synthetic gene designer	http://userpages.umbc.edu/~wug1/codon/sgd/	Wu et al. (2005)
GeneDesign	http://genedesign.org/	Richardson et al. (2006)
Gene Designer 2.0	http://www.dna20.com/resources/genedesigner	Villalobos et al. (2006)
OPTIMIZER	http://genomes.urv.es/OPTIMIZER	Puigbò et al. (2007)
Visual gene developer	http://www.visualgenedeveloper.net/	Jung and McDonald (2011)
Eugene	http://bioinformatics.ua.pt/eugene	Gaspar et al. (2012)
mRNA Optimizer	http://bioinformatics.ua.pt/software/mRNA-optimiser	Gaspar et al. (2013)
COOL	http://bioinfo.bti.a-star.edu.sg/COOL/	Chin et al. (2014)
D-Tailor	http://sourceforge.net/projects/dtailor/	Guimaraes et al. (2014)

In Table 2, we summarize major design objectives of each gene design tool. Several of the tools provide additional functionality not listed here, but mentioned in the following subsections.

**Table 2:** **Overview of gene design tools’ features**.

Gene design tool	Oligo generation	Rho-independent transcription terminator elimination	Prokaryotic ribosome binding site elimination	Codon usage	Codon context	mRNA secondary structure	GC/AT content	Restriction site manipulation	Motif avoidance	Repetitious base removal	Codon auto- correlation adjustment	Hidden stop codons	Hydropathy index optimization
DNAWorks	X			X				X					
Jcat		X	X	X				X					
Synthetic gene designer	X			X				X		X			
GeneDesign	X			X				X					
Gene Designer 2.0				X				X					
OPTIMIZER	X			X			X	X	X				
Visual gene developer				X		X	X	X	X	X		X	
Eugene				X	X	X	X	X		X	X	X	
mRNA Optimizer						X							
COOL				X	X		X	X	X	X		X	
D-Tailor				X		X	X	X					X

### DNAWorks

*DNAWorks* (Hoover and Lubkowski, 2002) is a web-based application that enables codon usage customization and oligo generation. It was originally created to automate the process of oligonucleotide design for synthetic gene construction. It includes functionality to adjust codon utilization, albeit with peculiar restrictions and an idiosyncratic codon assignment method. The user can customize codon usage via a frequency percentage threshold. The value of this threshold determines which synonymous codons will be substituted. For example, setting the frequency threshold to 20% allows only codons with representation greater than 20% of all instances of that particular amino acid to be viable substitutes. However, the program always considers the first and second most abundant codons to be viable candidates, independent of their absolute frequency. Therefore, maximizing the frequency threshold (100%) would force all amino acids to be coded by the top two most abundant codons. As such, one amino acid – one codon design is unattainable with DNAWorks. Once the optimization/oligo generation process commences, the software uses a randomized substitution heuristic strategy similar to simulated annealing. As codons are randomly mutated, changes are accepted or rejected based on a volatility (temperature) value. At higher volatilities, the program will be more likely to accept detrimental mutations while the converse is true at lower temperatures.

The codon optimization features of the tool supplement its oligo generation capabilities.

### Jcat

*Java Codon Adaptation Tool (Jcat)* (Grote et al., 2005) is a web-based application featuring codon usage optimization based on CAI score, restriction enzyme binding site elimination, rho-independent transcription terminator elimination, and prokaryotic ribosome binding site elimination.

Jcat optimizes target gene codon usage by maximizing its CAI score, which leads to designs that utilize a single codon for each amino acid. Reference sets of highly expressed genes for expression in a target host are retrieved from the PRODORIC database (Münch et al., 2003; Grote et al., 2009), which is maintained by the authors of the Jcat software. The methodology for generating codon usage data regarding highly expressed genes stored in the PRODORIC database is described in Carbone et al. (2003).

In addition to optimizing genes based on CAI score, Jcat allows users to eliminate certain restriction sites from a pre-defined list. The tool also offers options to avoid rho-independent transcription terminators and prokaryotic ribosome binding sites.

### Synthetic gene designer

*Synthetic Gene Designer* (Wu et al., 2005) is a web-based tool with the gene optimization functionality including codon usage optimization based on CAI score, restriction site elimination, repetitious segment elimination, and oligo generation.

The program allows the user to control the CAI score by setting an *optimality factor*, which varies from 0 to 64. Setting the optimality factor to *0* forces the program to utilize predominantly the most frequent codon for each amino acid, where the opposite effect takes place when the optimality factor is set to *64*, with intermediate values producing less polarized designs. Codon changes are performed randomly at any set value of the optimality factor, and each time the program is run produces a different design.

The program also provides for the elimination of unwanted restriction sites from a pre-compiled list of popular restriction enzymes (but only 29 in total), and the repetitious segments GGGGG/CCCCC and AAAAA/TTTTT. These modifications are performed once codon optimization has been concluded; restriction sites and repetitious segments are avoided at the expense of the synthetic gene’s codon usage. The program also allows the user to manually edit the gene. The synthetic gene can be easily regenerated with the same set of constraints (optimality factor, motifs to avoid, etc.) at the push of a button, facilitating user experimentation.

At the time it was created, Synthetic Gene Designer utilized the CUTG database (Nakamura et al., 2000), enabling gene optimization based on reference codon usage of any organism within GenBank. However, as of this writing, this feature is not functional. The program does make available through sets of highly expressed genes from model organisms, as referenced in studies such as Sharp and Li (1986), Carbone et al. (2003), and others.

### Gene design

*GeneDesign* (Richardson et al., 2006) is a web-based suite of tools/modules aiming to aid both the analysis and design of synthetic genes. Available modules can perform codon usage manipulation, restriction site incorporation and elimination, sequence analysis, and oligo generation.

The program offers a modular approach to gene design in which each individual modification (codon manipulation, restriction site addition/subtraction, etc.) is performed independently. In addition, the software comes with the option to successively manipulate a single gene through the selection of the “*Design a Gene*” method. In this case, the program automates the process of exporting modified genetic material from one module to the next. This affords the user the flexibility to manipulate a single target gene using any of the provided modules in any order.

Codon optimization can be performed with two modules: the codon juggling module and the back-translation module. Both methods only allow for a single codon to code for any particular amino acid. When optimizing through the back-translation module, this single codon will always be the most optimal codon, where using the *codon juggling* module the user can customize which codon is used. These customization options include a “next most optimal” design in which the second most optimal codon is used, and a “most different” design in which the codon most different from the optimal codon is used. Reference codon usage data come from Sharp et al. (1986).

### Gene designer

*Gene Designer* (Villalobos et al., 2006) is a stand-alone software that facilitates the construction of novel genetic material through an intuitive drag-and-drop approach to add and remove genetic elements. However, at its current state, the software lacks optimization functionality, which seems to have been available in previous versions. In order to optimize a gene, a quote must be requested from DNA 2.0, the company that created Gene Designer (DNA 2.0), and the optimized sequence must be purchased. As such, we were unable to test the claimed optimization functionality of Gene Designer. As it stands, the only features that come with Gene Designer upon download are those to manually build/edit a gene.

### OPTIMIZER

OPTIMIZER (Puigbò et al., 2007) is a web-based tool. Its functionality includes codon usage optimization based on CAI and ENc, restriction site elimination, motif avoidance, and oligo generation.

The software makes use of a pre-compiled set of codon usage statistics for about 150 genomes as the basis for its optimization. These reference sets consist of ribosomal proteins and other highly expressed genes within genomes that have been determined to be under translational selection, based on the RSCU codon evenness measure, which were created using a customized iterative process.

Codon usage in OPTIMIZER can be adjusted using three methodologies:

1. *CAI Optimal*: Every amino acid within the sequence is coded for by exactly one optimal codon (the most frequent in the group of highly expressed genes).
2. *Guided random*: Monte Carlo synonymous substitutions are performed to approximate the target codon distribution of the reference set of highly expressed genes.
3. *Selective optimization*: The user selects the number of least frequently used codons that should be eliminated from the sequence.

### Visual gene developer

*Visual Gene Developer* (Jung and McDonald, 2011) is a stand-alone tool utilizing modular optimization components, enabling user-accessible programing and addition of new functionality. The program implements an intuitive graphical user interface with a multitude of gene design options, which could prove overwhelming to the casual user. Optimization functions that are natively supported by the program include mRNA secondary structure and free energy prediction/optimization, CAI score optimization, and restriction site and other user-designated pattern elimination. The source code for inbuilt modules cannot be modified; however, Visual Gene Developer does support script programing using Visual Basic script or Java script for development of additional modules.

Modules with distinct functionality can be selected in any order to be applied toward the optimization of a gene. For example, one could designate to initially optimize the mRNA free energy, then use a Monte Carlo probabilistic algorithm to optimize the gene’s CAI (in a similar fashion to OPTIMIZER and Gene Designer), and finally silently remove undesirable restriction sites. Codon usage datasets used in CAI optimization are obtained from the CUTG database (Nakamura et al., 1999), where user-defined tables are provided as an option.

### COOL

*Codon Optimization OnLine* (Chin et al., 2014) is another web-based utility that can optimize for multiple objectives. Optimization functions that the program can perform include codon usage optimization based on CAI and Individual Codon Usage (ICU), codon context bias optimization, hidden stop codon optimization, G/C content adjustment, and restriction site and other pattern elimination.

Codon optimization based on ICU aims to modify the codon distribution to resemble a given reference set. The tool provides suggested reference gene sets for four organisms (without any information about the origin of the sets), and the user can customize reference genes manually.

The optimization process uses a genetic algorithm to produce several approximately Pareto-optimal solutions given a set of design criteria. Randomly generated sequences are evaluated, ranked, and mutated until a stability threshold is reached, at which point the fittest sequences based on the chosen properties are outputted and the algorithm terminates.

### Eugene

*Eugene* (Gaspar et al., 2012) is a stand-alone tool developed for multi-objective gene optimization. The program uses an intuitive user interface that is straightforward and easy to use. Eugene also automatically loads relevant database data (KEGG, NCBI databases) upon loading a gene to the workspace using identifier information provided in the gene’s FASTA/GenBank file. Optimization functions available through Eugene include hidden stop codon optimization, mRNA free energy optimization, codon usage *harmonization* based on CAI or RSCU, restriction site elimination, G/C content customization (provided as a percentage), codon autocorrelation adjustment, repetitious segment removal, and codon context bias optimization.

In our opinion, Eugene is one of the most versatile tools available for gene optimization; however, the program is accompanied with scarce documentation, obstructing our efforts to effectively interpret its output. Obfuscation of results is exacerbated by the use of “percentages” to indicate improvement toward a target objective, instead of widely accepted scores. Genes can be redesigned using either a rapid simulated annealing approach or a slower genetic algorithm that provides the user with several approximately Pareto-optimal solutions, one of which can be selected and uploaded to the workspace.

### mRNA optimizer

*mRNA Optimizer* (Gaspar et al., 2013) is a command-line stand-alone utility developed solely for the purpose of optimizing mRNA secondary structure free energy. The program itself is easy to use if one has a working understanding of operating the command line. The software is written in Java and is distributed as a jar archive.

As mentioned in Section “RNA Secondary Structure,” optimization of a protein-coding sequence for minimum free energy (MFE) has time complexity of O(*n*^3^) as a function of the sequence length *n* when maximizing MFE, and is NP-hard when minimizing. The mRNA Optimizer utilizes a simulated annealing heuristic to explore the RNA folding landscape, and uses a pseudo-MFE measure to approximate the Gibbs free energy. A simpler algorithm with quadratic complexity is employed to compute a pseudo-MFE, which correlates well with the actual MFE, by processing all single stem-loop conformations of the molecule during each iteration of the simulated annealing procedure.

### D-Tailor

*D-Tailor* (Guimaraes et al., 2014) is another stand-alone tool (written in Python) that employs multi-objective optimization and modularity in creation of synthetic genes. Optimization functions that the program natively supports include codon usage optimization based on CAI score, restriction site and other pattern elimination, G/C or A/T content optimization, mRNA secondary structure optimization, and hydropathy index optimization.

For an experienced Python programmer, D-Tailor is the most customizable gene synthesis tool of the ones considered; all other users will struggle to perform even a single optimization task. The source code is available for access and modification, unlike most other tools. On the other hand, in order to run a user-defined optimization, the user must add a Python class or edit an existing class to designate which features to use and to what extent they will be optimized. D-Tailor comes with template code to guide one’s efforts.

D-Tailor provides a generic class that can be extended to modify any criteria the user considers relevant. These features are optimized through the designation of levels that a sequence should exhibit after optimization. For example, one could divide the entire spectrum of CAI scores (0–1) into five levels (1: 0–0.2, 2: 0.2–0.4, etc.). By then setting the desired CAI level of the synthetic sequence, D-Tailor will generate solutions until a satisfactory sequence is found.

The optimization procedure of D-Tailor consists of two steps: sequence selection and sequence evolution. In the first step, a template sequence is retrieved from the repository of sequences, a MySQL database that is populated as the algorithm iterates (on first iteration, the database contains only the seed sequence) based on the optimization objectives. This selected sequence is then synonymously mutated a number of times (default 100) until a sequence exhibiting preferential target property levels is generated. This mutation process starts by analyzing the current sequence’s fitness in each relevant property and identifying one property that needs improvement. The sequence is then modified toward the improvement of this property (adhering simultaneously to any user-designated avoidance features such as restriction sites, motifs, promoter sequences). The algorithm then computes the Euclidian distance of the modified sequence relative to the given design target to determine whether improvement was made toward the optimal solution, in which case the new sequence is added to the database. The process can be customized using three user-selected options:

1. Direction optimization: solely accept sequences with smaller Euclidian distances to the target.
2. Neutral optimization: accept modified sequence based on defined probabilities.
3. Temperature-based optimization: probability of choosing less favorable sequences decreases as successive iterations of the algorithm are performed (similar to simulated annealing).

### Other tools

In addition to the tools listed in the previous subsections, a number of additional software applications have been created to aid gene redesign and customization for synthesis. Few of these tools were unattainable at the time of this study. These include *Upgene* (Gao et al., 2004), *Gene Morphing System (GeMS)* (Jayaraj et al., 2005), and *Gene Composer* (Lorimer et al., 2009). The basic functionality of these programs can be largely found in other currently available tools.

## Gene Design Tools in Practice

We downloaded, installed, configured, and run all stand-alone gene design programs, and configured and used all web-based tools that were detailed in the previous section. To test their functionality, we redesigned a copy of the *Aequorea victoria* GFP gene, with GenBank Accession Number M62653, for heterologous expression in *Escherichia coli*. We used the *E. coli* strain K12 for all experiments where we had to provide coding sequences, and for all comparisons.

All programs except for Visual Gene Developer were tested on a desktop computer with an Intel Haswell i7-4770 CPU (22 nm lithography) running at 3.4 GHz, with 16 GB of main memory, and Ubuntu 14.04 operating system. The Visual Gene Developer tool was tested on a laptop equipped with an Intel Haswell i7-4700MQ CPU (22 nm lithography) running at 2.4 GHz, with 8 GB of main memory, and MS Windows 8.

We tested all basic functionality of the programs, and most of the optimization features and combinations provided. We are reporting our results and impressions on a subset of these features, for the following reasons:

- Functionality of all tools for most optimization objectives works as described in corresponding documentation. We attempt to note discrepancies instead of reporting conformity. Codon usage optimization in one amino acid – one codon design produces consistently the expected results.
- Many optimized designs cannot be effectively evaluated for accuracy or efficiency, since they are created based on unique objective measures of each specific tool, the tools use customized reference sets of genes, or heuristics are employed, which are not comparable between tools. Heuristics implemented in the majority of tools often generate suboptimal results, which cannot be compared to unattainable optimal solutions.

We chose to present experimental results for the following design objectives:

- Codon usage optimization for heterologous expression, based on a target distribution.
- RNA Gibbs free energy optimization.
- Restriction site elimination.

In addition, we report our personal experiences installing, customizing, and running the software (where applicable). We describe a tool as *unresponsive* when the time period to optimize a gene toward a set of objectives surpasses half an hour without producing a design or updating a progress indicator.

### Ease of installation and use

Table 3 displays the form of availability of each tool (web based or stand-alone), the operating system where it can be accessed, the availability of its source code, the ease of installation, and overall ease of use. The reference point for the last two subjective measures is a confident user of the listed operating systems (MS Windows, Linux, and Mac OS X), with limited computer programming experience, moderate Information and Communication Technologies (ICT) background, and moderate experience in program installation and terminal command-line usage in Un*x systems.

**Table 3:** **Availability and ease of installation/use of computational gene design tools**.

Gene design tools	Availability	Operating system	Implementation language	Source code availability	Ease of installation	Ease of use
DNAWorks	Web based	Any	Fortran90	N/A	N/A	Easy
Jcat	Web based	Any	Java	N/A	N/A	Easy
Synthetic gene designer	Web based	Any	PHP, Javascript, and Perl	N/A	N/A	Medium
GeneDesign	Web based	Any	Perl and C	N/A (github repository unavailable)	N/A	Easy
Gene Designer 2.0	Stand-alone	Mac OS X, Windows, Linux	Unknown	N/A	Easy	Medium
OPTIMIZER	Web based	Any	PHP	N/A	N/A	Medium
Visual gene developer	Stand-alone	Windows	.Net Framework	Partially available^a^	Easy	Medium
Eugene	Stand-alone	Mac OS X, Windows, Linux	Java	N/A	Easy	Easy
mRNA Optimizer	Stand-alone, command line	Mac OS X, Windows, Linux	Java	N/A	Easy	Medium
COOL	Web based	Any	Perl (functional back-end)	Partially available^b^	N/A	Medium
D-Tailor	Stand-alone	Mac OS X, Windows, Linux	Python	Available	Hard	Hard

*^a^Visual gene developer allows users to develop new optimization modules, but not edit existing modules*.

*^b^The source code of COOL is partially available for modification. Certain parts of the code are only provided as machine executable binary files*.

*The terms “easy,” “medium,” and “hard” are subjective and refer to the ease of installation and use of the tools by a user with limited to moderate ICT and programing skills*.

**Figure 1:**
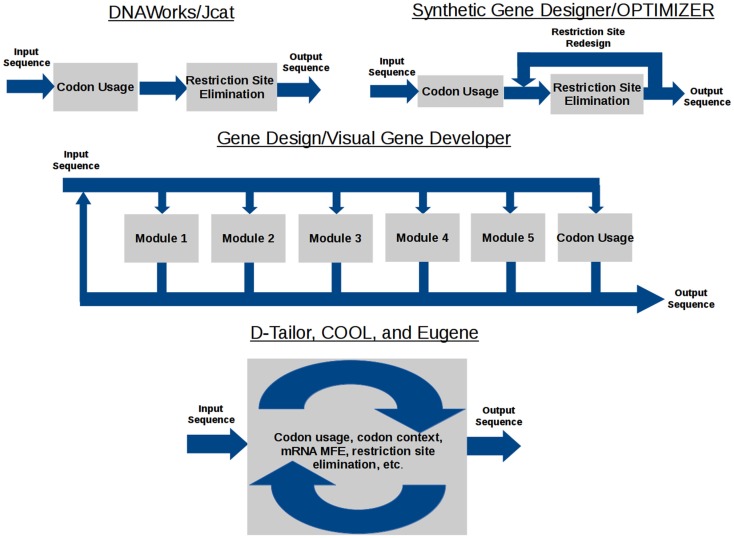
**Gene design software data flow**.

The data flow model of each gene design tool is shown in Figure 1. DNAWorks, Jcat, Synthetic Gene Designer, and OPTIMIZER run optimization tasks sequentially, with the potential of each successive optimization running antagonistically against preceding tasks. Visual Gene Developer and Gene Design offer modules each providing an individual optimization, but can also suffer from antagonism. These modules can be used in any user-designed order and frequency. Eugene, COOL, and D-Tailor optimize all objectives concurrently, utilizing metaheuristics.

### Codon optimization tests

**Figure 2:**
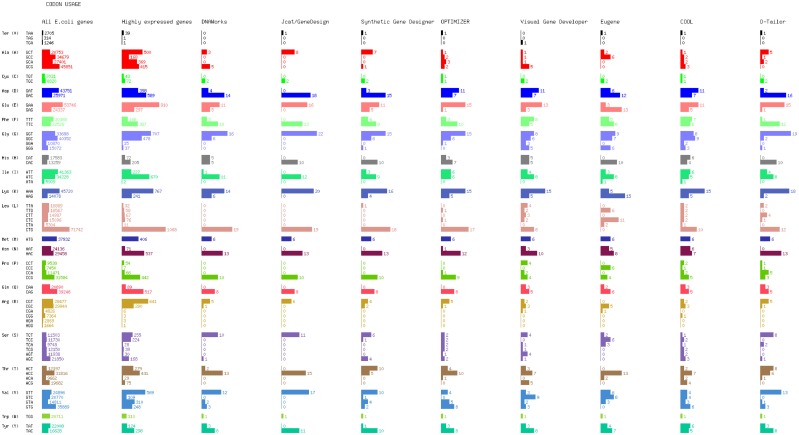
**Codon usage distributions of gene designs generated by optimization tools**.

We redesigned the GFP gene encoding for expression in *E. coli*, based on codon usage optimization. We selected to randomize the synonymous substitutions for each program that provides such an option, and to pursue emulation of the target distribution when available, instead of maximization of CAI, since the latter exclusively utilizes a single codon for each amino acid. Results are displayed in Figure 2, where the first column displays the cumulative codon distribution of all *E. coli* genes (strain K12), and the second shows the cumulative codon distribution of all *E. coli* highly expressed genes, as determined by Sharp and Li (1986).

#### DNAWorks

By observing the optimized codon distribution, it is not obvious that DNAWorks uses the two most abundant codons for the majority of synonymous substitutions. This could be attributed to utilization of a different reference set than the one displayed, the *E. coli* class II genes, which are highly and continuously expressed during exponential growth, as determined by Medigue et al. (1991). Another possible explanation is the use of a simulated annealing procedure to decide synonymous changes, which adds an element of randomness.

#### JCat and GeneDesign

The “one amino acid – one codon” approach is clearly evident in our results. The synthesized gene in this test case had a CAI of 1, since codon usage was the single target of this optimization.

#### Synthetic gene designer

Codon usage closely resembles the distribution of highly expressed *E. coli* genes. For the displayed design, the optimality factor of the program was set to 1. The GFP gene was optimized based on a reference set defined in Carbone et al. (2003), which may have produced some of the observed variation.

#### OPTIMIZER

Selecting the *Guided Random* option for optimizing codon usage, OPTIMIZER generates a design with a codon distribution that differs in several amino acids from the reference sets displayed. OPTIMIZER has pre-compiled its own reference datasets of highly expressed genes based on RSCU evenness. Codon usage for several amino acids resemble the Sharp/Li set distribution, where for other amino acids there is a close resemblance to the overall *E. coli* gene distribution. After running a number of optimization cycles with the guided random method (results not shown), OPTIMIZER appear to vary widely its codon usage patterns between designs, occasionally utilizing under-represented codons in the reference set more frequently than overrepresented ones.

#### Visual gene developer

Codon utilization generally reflects the overall *E. coli* codon usage, which is supported by the tool’s dependence on the CUTG database to obtain reference sets. Certain variation could be possibly explained by the use of a Monte Carlo randomized algorithm for synonymous mutations.

#### Eugene

To test Eugene’s codon optimization capabilities, we selected the *harmonization method based on CAI* option. The resulting codon usage design does not resemble any of the reference datasets that other programs are using. Successive iterations of the same optimization method using simulated annealing always produce the same result, which leads us to believe that the discrepancy is systematic. Eugene’s documentation mentions that reference datasets are retrieved from KEGG and PDB, but specifics on the selections are absent.

#### COOL

We chose the option to maximize codon usage based on ICU. The method COOL uses to create customized reference sets is not documented, but the tool makes available the list of genes used as a reference, and allows the user to customize it. Using the built-in reference set, we generated an optimized design whose codon distribution resembles the overall *E. coli* usage.

#### D-Tailor

We performed codon optimization based on CAI, with a target score range of 0.7–0.95. The actual score obtained after optimization was approximately 0.7. The resulting codon distribution resembles the highly expressed gene set of Sharp and Li, which is the reference set that D-Tailor is using as well. However, should one wish to optimize for expression in another host, he/she would have to manually generate this same information from their own set of highly expressed genes and make necessary edits within the source code.

#### Comments

The Eugene tool becomes unresponsive when optimizing for a single objective using the evolutionary heuristic, for which reason it was not tested. D-Tailor becomes unresponsive or fails to terminate when target score ranges are set unrealistically, although it is hard to determine *a priori* which ranges would be realistic for each objective. All other programs run efficiently and generated results as expected.

### mRNA free energy test

Four of the gene design tools are capable of optimizing the mRNA folding energy of a given gene. Of these, Eugene, mRNA optimizer, and D-Tailor provide the option to maximize or minimize the MFE of the entire gene; the latter two tools also allow for the specification of a region of the gene to perform that optimization. Visual gene developer allows the user to specify target MFE for a window of a given size, and attempts to optimize each window along the sequence independently.

We used the UNAFold utility to compute the folding energy of the wildtype GFP encoding, which was determined to have an MFE of −133.80 kcal/mol. We then run mRNA MFE maximization and minimization design experiments for the GFP protein, with results summarized in Table 4.

**Table 4:** **MFE optimization experiments of GFP mRNA**.

Tool	Minimized MFE (kcal/mol)	Maximized MFE (kcal/mol)	Running time (s)	
			Maximization	Minimization
Eugene	−202.20	−74.40	8.4	8.4
mRNA Optimizer	−133.80	−76.67	0.0	10.2
D-Tailor	−190.40	−99.04	144.5	142.0

All programs were configured to pursue a single objective. The Eugene tool manages to produce the most extreme designs. The mRNA optimizer performs equally well when maximizing the MFE, but returns the mRNA sequence without modifications when minimizing the MFE.

### Restriction site removal tests

As we discussed in Section “Restriction Sites, Hidden Stop Codons, and Other Motif Avoidance,” incorporating and eliminating restriction sites and other patterns can lead to hard computational problems, even in the absence of other optimization objectives. It is rare though for such cases to occur in practice. When the patterns do not overlap and other optimization objectives do not interfere, pattern incorporation and elimination are simple optimization tasks that all gene design tools under consideration perform with ease.

To provide a useful test of the tools’ flexibility, and to inform the user of the potential behavior of the programs under competing optimization objectives, we devised a test case that leads to an unattainable design, where a compromise will have to be made in order to generate a solution. This test case involves the design of a GFP mRNA encoding that does not contain any *Nco*I (CCATGG) and *Nde*I (CATATG) restriction sites, while encoding every occurrence of the amino acid Proline with codon CCA, and Histidine with CAT. The CATATG pattern occurs at position 166 of wildtype GFP, and CCATGG appears at position 230. Only a synonymous substitution of CAT can eliminate the first site, and similarly a synonymous substitution of CCA can eliminate the second. The results from running this test case on each individual program are presented below.

DNAWorks claims to eliminate the restriction sites, but the sites were still present in the output.

JCat does not provide functionality to specify which codons should code for which amino acid. Additionally, the user cannot alter the codon usage of the reference set.

GeneDesign can be customized to encode any amino acid with a single codon. The sequential optimization of the objectives results in codons being synonymously substituted to avoid incorporation of the restriction sites.

Synthetic Gene Designer becomes unresponsive when provided these specifications.

OPTIMIZER overwrites the codons to avoid the restriction site.

Visual Gene Developer claims to eliminate the restriction site but outputs a design that still contains the restriction sites.

Eugene does not offer the functionality to alter the codon usage data that are used during optimization.

COOL uses restricted codons for Proline and Histidine to eliminate the restriction sites.

D-Tailor required code modification to get the experiment to work. Even so, once the codon table was modified to only utilize CCA to code for Proline, the program would not accept the input sequence, because GFP already contains the restriction site. It appears that D-Tailor is not actively modifying optimized sequences to remove restriction sites, but merely guarantees that a restriction site will not be added to the existing sequence. The program terminates if one forbidden site is already present.

## Discussion

Synthetically designed genes have historically been optimized for host codon bias and mRNA secondary structure in order to maximize gene expression in the host. However, many studies show that these are not necessarily the “main” forces acting on translation throughput. It is not imminently clear which forces of codon selection are stronger than others and which are artifacts of others; moreover, experiments relying on random mutation often do not provide concrete answers to these questions. With the decrease in price of *de novo* gene synthesis, hypothesis-driven studies using reverse genetics are expected to rise as the preferred method to design more controlled experiments. In addition to understanding the forces behind natural gene design more thoroughly, rational gene design may also improve heterologous gene expression and metabolic pathway optimization.

Our study involved numerous tools that enable the rational customization of protein-coding genes for experimentation concerning expression. Each tool has its own strengths and limitations. Newer tools such as Eugene, COOL, and D-Tailor now provide most of the functionality of older ones, but often have steeper learning curves and more complicated options and interfaces. Web-based applications are the easiest to access and fastest to learn, where multi-objective optimization tools often need to come as stand-alone programs to make use of the computational power of a local workstation. No tool is perfect or suited for every optimization task and user experience level, but synthetic biologists today have access to a considerable arsenal of flexible and capable design tools, which can be effectively used to design the next generation of synthetic constructs for hypothesis testing.

There exists much space for improvement in the gene design software domain. With the exception of Eugene, most recent tools sacrifice ease of use for multi-objective optimization. When single optimization objectives lead to hard computational problems, or the tools are optimizing protein-coding regions toward multiple objectives, current tools do not provide guarantees on solution optimality, and most do not guarantee even solution quality. No tool examined in this review provides any form of quantifiable expression predictive power based on design decisions such as codon and codon context choices, or mRNA structure manipulation (in contrast to RBS design tools briefly mentioned in Section “Ribosomal Binding Site”). This is a direct consequence of the lack of extensive wet lab experimentation to determine the contributions of individual codons, codon pairs, etc. when expressing genes in different organisms. New gene design tools or updated versions of existing tools would benefit from implementation of additional features such as splice site and other pattern incorporation/elimination as described by position weight matrices and other probabilistically based methods, dinucleotide distribution manipulation, and other factors posited to affect gene expression. In addition, current tools have not explicitly addressed optimizations toward important gene design objectives other than translation rates and restriction site placement, such as mRNA stability. Toward these goals, modular architectures adopted by tools such as D-Tailor, which allow experienced Python programmers to incorporate additional optimization objectives, may provide the necessary flexibility to enable tool evolution and wide adoption by the gene design community.

## Conflict of Interest Statement

The authors declare that the research was conducted in the absence of any commercial or financial relationships that could be construed as a potential conflict of interest.

## Footnotes

^1^The big-O notation is used to describe upper bounds on the growth rate of functions. In this case, a time complexity of O(*n*65) indicates that the running time of the algorithm will be roughly proportional to a 65th order polynomial function of the size of the input, in the worst case. As the input size increases, in this case the length of an RNA molecule, the time it takes to compute its optimal codon pair bias score increases roughly proportional to the 65th power of the molecule length.

^2^NP-hard are algorithmic problems for which no one currently knows whether they can be solved in polynomial time, meaning that the time we need to solve such a problem for an input of size *n* is expected to be an exponential function of *n* in the worst case. Such problems are considered hard to solve optimally for any but the smallest input instances.
